# The *Eucalyptus* terpene synthase gene family

**DOI:** 10.1186/s12864-015-1598-x

**Published:** 2015-06-11

**Authors:** Carsten Külheim, Amanda Padovan, Charles Hefer, Sandra T Krause, Tobias G Köllner, Alexander A Myburg, Jörg Degenhardt, William J Foley

**Affiliations:** Research School of Biology, College of Medicine, Biology and the Environment, Australian National University, Canberra, 0200 Australia; Department of Botany, University of British Columbia, Vancouver, BC V6T1Z4 Canada; Institut für Pharmazie, Martin-Luther Universität Halle-Wittenberg, 06120 Halle, (Saale) Germany; Department of Biochemistry, Max Planck Institute for Chemical Ecology, 07745 Jena, Germany; Department of Genetics, Forestry and Agricultural Biotechnology Institute, Private Bag X20, Pretoria, 0028 South Africa

**Keywords:** *Eucalyptus*, Myrtaceae, Terpene synthase, Essential oil, Monoterpenes, Sesquiterpenes, Evolution, Biodiversity, Herbivory

## Abstract

**Background:**

Terpenoids are abundant in the foliage of *Eucalyptus*, providing the characteristic smell as well as being valuable economically and influencing ecological interactions. Quantitative and qualitative inter- and intra- specific variation of terpenes is common in eucalypts.

**Results:**

The genome sequences of *Eucalyptus grandis* and *E. globulus* were mined for terpene synthase genes (TPS) and compared to other plant species. We investigated the relative expression of TPS in seven plant tissues and functionally characterized five TPS genes from *E. grandis*. Compared to other sequenced plant genomes, *Eucalyptus grandis* has the largest number of putative functional TPS genes of any sequenced plant. We discovered 113 and 106 putative functional TPS genes in *E. grandis* and *E. globulus*, respectively. All but one TPS from *E. grandis* were expressed in at least one of seven plant tissues examined. Genomic clusters of up to 20 genes were identified. Many TPS are expressed in tissues other than leaves which invites a re-evaluation of the function of terpenes in *Eucalyptus*.

**Conclusions:**

Our data indicate that terpenes in *Eucalyptus* may play a wider role in biotic and abiotic interactions than previously thought. Tissue specific expression is common and the possibility of stress induction needs further investigation. Phylogenetic comparison of the two investigated *Eucalyptus* species gives insight about recent evolution of different clades within the TPS gene family. While the majority of TPS genes occur in orthologous pairs some clades show evidence of recent gene duplication, as well as loss of function.

**Electronic supplementary material:**

The online version of this article (doi:10.1186/s12864-015-1598-x) contains supplementary material, which is available to authorized users.

## Background

*Eucalyptus* dominates Australian forests and woodlands and is truly the “essence” of Australia as well as being the dominant hardwood plantation tree in the world. High growth rates make eucalypts a desirable hardwood plantation tree for pulp, sawmills and biofuels. Foliar terpenes give eucalypts their characteristic odour, they are industrially important and mediate many ecological interactions. Several *Eucalyptus* species are used to produce *Eucalyptus* oil, a 1,8-cineole-dominated terpenoid mixture with antiseptic effects which is utilised in pharmaceuticals and as a scent and flavour. Eucalyptus terpenes also act to mediate ecological interactions including deterrents to insect herbivory [1,2], attractants [3] and repellents to vertebrate herbivores [4], cues to other toxic constituents [5], mediators of resistance to fungal infection [6], allelopathic agents [7], attractants for parasitoids and pollinators [8], determinants of leaf litter decomposition rates [9,10], mitigators to heat stress [11], as well as significant contributors to biogenic hydrocarbons in cities [12].

Although no terpenes are found exclusively in eucalypts, striking variation can be observed in the foliar terpene profile within a single species [13] or even within individual branches of a single tree [14]. Here we show that this variation is built on the largest family of terpene synthase genes of any plant yet sequenced.

Terpenes are formed from C_5_ precursors, which are produced either through the methylerithritol phosphate pathway (MEP) in the chloroplast or the mevalonate pathway (MVA) in the cytosol [15]. Geranyl pyrophosphate (GPP - C_10_) or geranylgeranyl pyrophosphate (GGPP - C_20_) are formed in the chloroplast, and farnesyl pyrophosphate (FPP - C_15_) in the cytosol. These prenyl pyrophosphates are the substrate for terpene synthases, which catalyse the production of mono-(C_10_) from GPP or di-(C_20_) terpenes from GGPP as well as hemiterpenes (C_5_) directly from isopentyl pyrophosphate in the chloroplast, or sesquiterpenes (C_15_) from FPP in the cytosol. The products of these multi product enzymes can be further modified by oxygenation by cytochrome P450 monooxygenases, or methylation by methyl transferases to form additional compounds.

The terpene synthase gene family (TPS) has been divided into three classes and seven sub-families: Class I consists of TPS-c (copalyl diphospate and ent-kaurene), TPS-e/f (ent-kaurene and other diterpenes as well as some mono- and sesquiterpenes) and TPS-h (*Selaginella* specific); class II consists of TPS-d (gymnosperm specific) and class III of TPS-a (sesquiterpenes), TPS-b (cyclic monoterpenes and hemiterpenes) and TPS-g (acyclic monoterpenes) [16]. These clades have been identified through sequencing and functional studies of a wide range of plants (*Arabidopsis thaliana*: [17], *Vitis vinifera*: [18], *Solanum lycopersicum*: [19], *Selaginella moellendorfii:* [20], *Populus trichocarpa:* [21]).

Often, plants that emit or store few terpenes, have only a small number of TPS genes, such as *Arabidopsis thaliana* (32 putative functional and 8 pseudo TPS genes: [17]) and poplar (38 putative functional genes: [21]). Other plants that have a more complex blend of terpenes, which are often stored in trichomes or other glandular structures, tend to contain a larger number of TPS genes (e.g. grape with 69 putative functional and 63 pseudo TPS genes [18]). Eucalypts contain a high diversity of terpenes, which are stored mainly in schizogenous secretory cavities in the leaf and flower buds [22]. Given the striking variation in terpenes that can occur within a single *Eucalyptus* species, we hypothesise that eucalypts will contain a highly diverse and abundant family of terpene synthase genes which can be differentially regulated to generate unique terpene profiles in different parts or at different times of the plant as required. We describe the *Eucalyptus grandis* TPS gene family from the recently sequenced reference genome of *E. grandis* (V1.1 annotation, www.phytozome.net) [23] and also make comparisons with a second species of *Eucalyptus* that has been sequenced (*E. globulus*) to understand how this gene family is arranged and how it has evolved over the relatively short time (ca. 12 million years) that these two species separated (Thornhill, Külheim and Crisp unpublished) and how it has contributed to the success of the genus *Eucalyptus* across its native and introduced range.

## Results

### Discovery of putative TPS genes from the *E. grandis* genome

We identified 172 loci in the *Eucalyptus grandis* genome with a high sequence similarity to known terpene synthase genes from other species. Loci that spanned fewer than three exons (out of seven for most TPS sub families) were not further considered which resulted in the exclusion of 20 loci. Loci were considered putatively functional if they were full length (with the exception of genomic areas where sequence data was missing), which had fewer than two frame shifts and stop codons (combined) or showed evidence of gene expression either through the presence of expressed sequence tag (EST) models or RNAseq data that matched the loci (Additional file 1: Table S1). Publically available RNAseq data (www.phytozome.net), as well as our own RNAseq dataset, were used to validate the genomic sequences. The resulting list of genes was then classified as (i) full-length, expressed with no premature stop codons or frame shifts (89), (ii) full-length, expressed with up to two stop codons or frame shifts (23), (iii) full-length, with no expression and no frame shift or stop codon (1), (iv) pseudogenes with more than two frame shifts or stop codons (39) and (v) partial genes (20) (Additional file 1: Table S2). A total of 113 loci in groups (i) (ii) and (iii) were considered to be putatively functional TPS genes (Additional file 1: Table S1).

**Figure 1:**
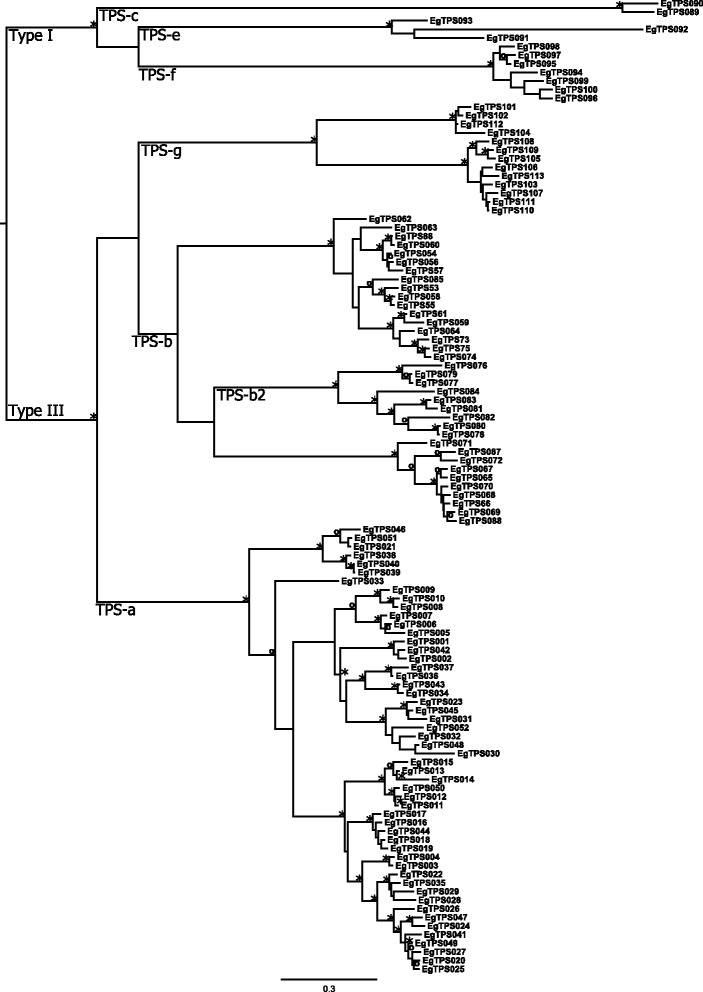
Phylogeny of the 113 putatively functional terpene synthase (TPS) genes in *Eucalyptus grandis*. Maximum likelihood analysis of the *E. grandis* TPS gene family rooted at the branching of type I and III TPS genes. Clade TPS-b2 contains acyclic terpene synthases from the rosids with main products of isoprene or ocimene. Bootstrap values supported by ≥ 80% are designated * while those with bootstrap values ≥ 95% are designated ^**O**^. Subfamilies are described on the branches.

Six of the eight currently recognized TPS subfamilies are present in *E. grandis*, the exceptions being the Gymnosperm specific TPS-d subfamily and the *Selaginella moellendorffii* specific TPS-h subfamily (Figure 1). Gene copy numbers in diterpene synthase genes (TPS-c and –e) were similar to those found in other plant species (Table 1). However, the other TPS subfamilies are much more abundant in *E. grandis* compared to other plant species (Table 1).

**Table 1:** **Gene copy numbers of TPS genes from plant species with a fully annotated genome separated by subfamilies**

	**Terpene type**	***E. grandis***	***E globulus***	***V. vinifera***	***P. trichocarpa***	***A. thaliana***	***S. lycopersicum***	***S. bicolor***	***O. sativa***	***S. moellendorffii***	***P. patents***
TPS-a	sesqui	52	45	29	13	23	12	15	19	0	0
TPS-b1	mono	27	28	8	10	6	8	2	0	0	0
TPS-b2	isoprene/ocimene	9	10	2	2	0	0	0	0	0	0
TPS-c	di	2	2	2	2	1	2	1	3	3	2
TPS-e	mono, sesqui, di	3	2	1	2	1	5	3	9	3	0
TPS-f	mono, sesqui, di	7	9	0	1	1	0	0	0	0	0
TPS-g	mono, sesqui, di	13	10	15	2	1	2	3	1	0	0
TPS-h	di	0	0	0	0	0	0	0	0	8	0
Total		113	106	57	32	33	29	24	32	14	2

Gene copy numbers differ between studies; numbers shown here are derived from the downloaded sequences from each species.

### Putative terpene synthases in *E. globulus*

We discovered 106 terpene synthase genes in *E. globulus* using a strategy similar to that applied to *E. grandis*, but without any gene expression data due to the lack of such datasets (Table 1). We further discovered 37 putative pseudogenes which contained multiple stop codons or frame shifts (Additional file 1: Table S3). Overall, the differences in gene copy numbers between *E. globulus* and *E. grandis* were small, with slightly fewer sesquiterpene synthases (TPS-a), two more mono- and hemi-terpene synthases (TPS-b) as well as TPS-f copies, similar copy numbers in TPS-c and –e and three fewer copies in the TPS-g subfamily (Table 1). Most pseudogenes were found among the TPS-a subfamily (16), with slightly fewer in the TPS-b (13) and four each in the TPS-f and –g subfamilies (Additional file 1: Table S3).

### Genomic organization of terpene synthase genes from *Eucalyptus grandis*

**Figure 2:**
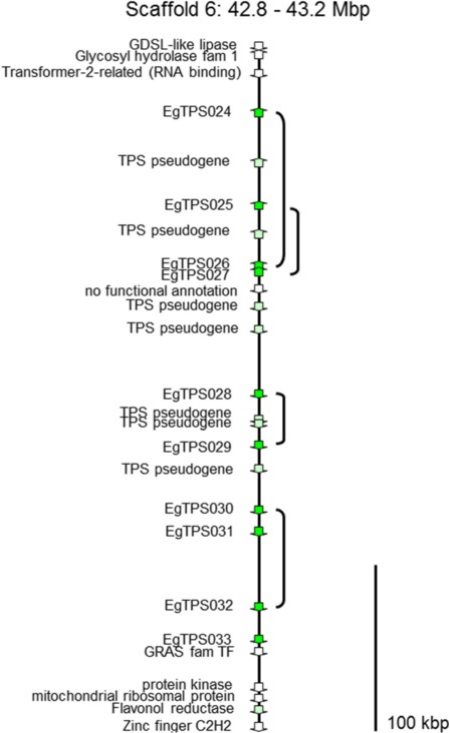
Genomic organization of a 400 kb multi-gene cluster from *Eucalyptus grandis*. A cluster of 10 putative functional (green arrows) and seven TPS pseudogenes (light green arrows) as well as other genes (white arrows) in the area are shown. Close phylogenetic relationships are indicated by brackets.

Most *E. grandis* TPS genes are organized in moderate to large groups (tandem gene arrays) in the genome. Each group contains only genes of the same TPS subfamily and genes are found in dense clusters. In some cases, the distance between two EgranTPS genes is less than 1 kb, e.g. between EgranTPS026 and EgranTPS027, the distance is only 508 bp between stop and start codon. On scaffold 6, there are 17 TPS genes and pseudogenes found within 317 kb, one TPS gene for every 18.6 kb (Additional file 1: Table S4 and Figure 2). The clustering of putative isoprene/ocimene synthases is even greater and on scaffold 11, eight isoprene synthase genes are found within 107 kb – one gene per 13.4 kb (Additional file 1: Table S4).

Genes from different steps in the terpene biosynthetic pathway are found nearby the clusters of *E. grandis* TPS genes, such as a prenyl transferase on scaffold 11 (Additional file 1: Table S4). The TPS-b cluster on scaffold 4 is also co-incident with eight quantitative trait loci (QTL) for foliar monoterpenes from *Eucalyptus nitens* and one for foliar sideroxylonal discovered by Henery and co-workers [24]. Other genes of plant secondary metabolism are also found close to clusters of terpene synthases (e.g. phenylalanine ammonia lyase, anthocyanidin reductase, flavonol reductase) as are many transcription factors (Additional file 1: Table S4).

**Figure 3:**
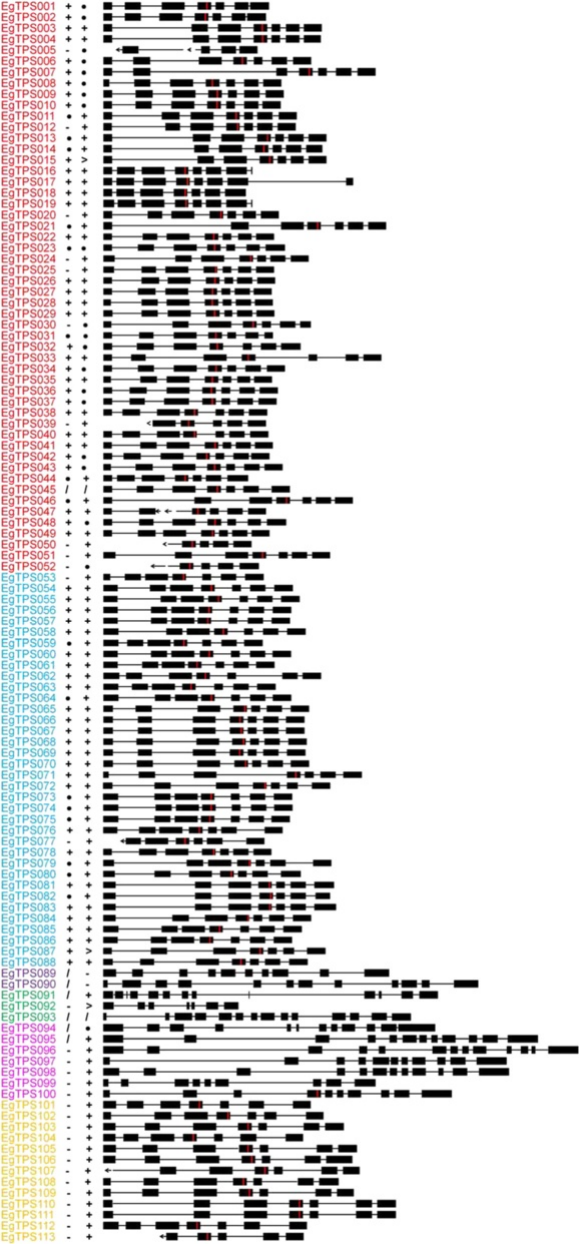
Gene structure and motif representation of putative functional terpene synthases from *Eucalyptus grandis*. Exon-intron structures were manually determined by comparison to previously characterized genes. Exons are shown by full boxes, while introns are depicted as lines. Arrows show missing genome sequences and the red line shows the location of the DDxxD motif. The presence and quality of conserved motifs (i) RR(x)_8_W and (ii) NSE/DTE are indicated after the gene name where “+” is a perfect motif match, “•” has one amino acid changed, “>” two amino acids changed and “–” means that the motif is not present. Gene names are coloured according to subfamily; TPS-a (EgranTPS001 – EgranTPS052) is red, TPS-b (EgranTPS053 – EgranTPS088) is blue, TPS-c (EgranTPS089 – EgranTPS090) is purple, TPS-e (EgranTPS091 – EgranTPS093) is green, TPS-f (EgranTPS094 – EgranTPS100) is pink and TPS-g (EgranTPS101 – EgranTPS113) is yellow.

Most (82 out of 92) EgranTPS genes of subfamilies –a, −b and –g, contain seven exons, with the exception of three TPS-a genes (EgranTPS016, EgranTPS017 and EgranTPS019) which appear to have recently obtained a seventh intron near the 3′ end of exon seven and seven genes for which sequence data is incomplete (Figure 3). Genes from the remaining subfamilies, −c, −e and -f, contain between 9 and 14 exons, with the exception of EgranTPS92 which has just seven exons. The length of the introns is largely conserved with a few exceptions, notably intron 1, which is highly variable across subfamilies -a, −b, and -g (Figure 3).

### Global expression profiling of terpene synthase genes from *Eucalyptus grandis*

**Figure 4:**
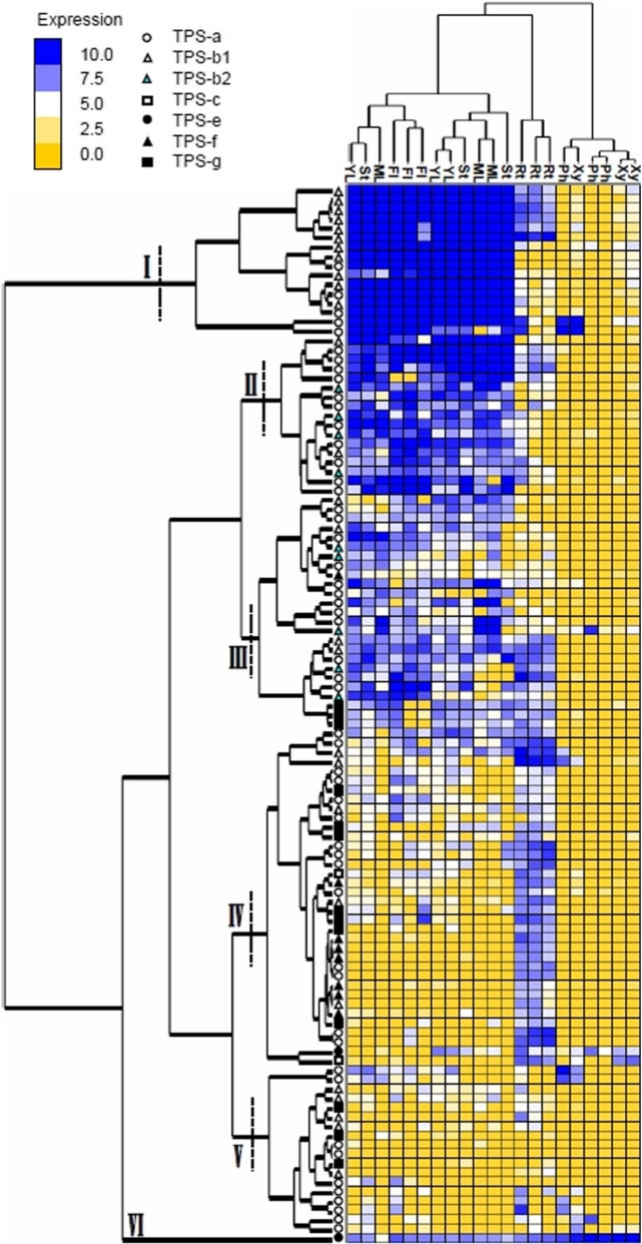
Gene expression of 113 terpene synthase (TPS) genes from *Eucalyptus grandis* in seven plant tissues. The log2 expression values determined from FPKM values of RNAseq data are shown. Cluster analysis of tissues and genes was performed and the *E. grandis* TPS subfamilies are indicated. The tissues investigated are: YL: young leaf, ST: shoot tips, ML: mature leaf, Fl: flower, Rt: root, Ph: phloem and Xy: xylem.

A heat map showing relative transcript abundance of 113 TPS genes in three biological replicates of seven tissues is shown in Figure 4. The hierarchical cluster analysis of the tissues shows that the six wood tissue samples (immature xylem and phloem) form one cluster, three root tissues a separate cluster and the ‘green tissue’ comprised of young leaf, mature leaf, shoot tips and flowers a third cluster. The only two tissues where all three biological replicates cluster together are root and flower. The hierarchical clustering of genes shows a cluster of highly expressed genes in the ‘green tissue’, dominated by TPS-b1 genes (I). The next major cluster is also expressed mostly in ‘green tissue’, but to a lesser extent (II). Gene expression cluster II contains mostly genes from the TPS-a group with several TPS-b1 and -b2 genes. Cluster (III) is moderately expressed in ‘green tissue’ with one sub-cluster expressed in root as well. Genes that cluster by gene expression patterns are a mixture of most subfamilies, excluding TPS-c and -e. Cluster (IV) shows highest levels of expression in root tissue, with minor expression in ‘green tissue’ and wood tissue. All subfamilies bar isoprene/ocimene syntases (TPS-b2) are represented in this cluster. Cluster (V) has the overall lowest level of gene expression scattered over all tissues, containing genes from TPS-a, −b1 and -g. Finally, cluster (VI) is a single gene, which is expressed constitutively in all tissues at moderate to high levels. This gene encodes a putative ent-kaurene synthase, which produces the precursor for gibberellic acid.

### Phylogenetic analysis of *Eucalyptus* TPS genes in comparison to other plant species

**Figure 5:**
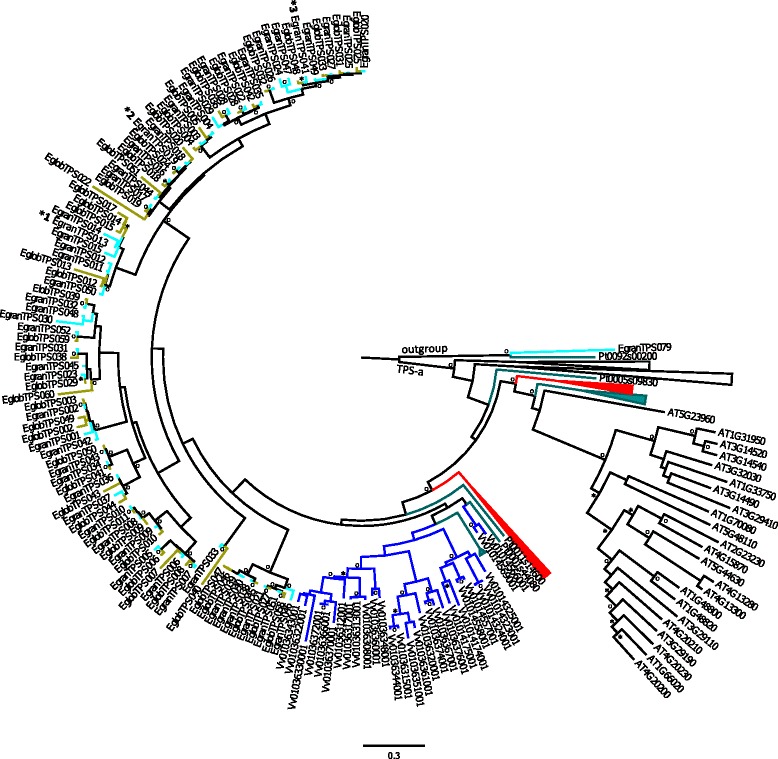
Phylogeny of the TPS-a subfamily. Maximum likelihood analysis of TPS-a subfamily from *E. grandis* and *E. globulus* in comparison to *Vitis vinifera* and *Arabidopsis thaliana*. Functionally characterized enzymes are indicated with an * with main products: *1 isoledene / cadinene, *2 germacrene D, *3 bicyclogermacrene. Bootstrap values supported by ≥ 80% are disignated * while those with bootstrap values ≥ 95% are designated ^**O**^. Two TPS-b genes from *E. grandis* and *P. trichocarpa* were selected as outgroups.

**Figure 6:**
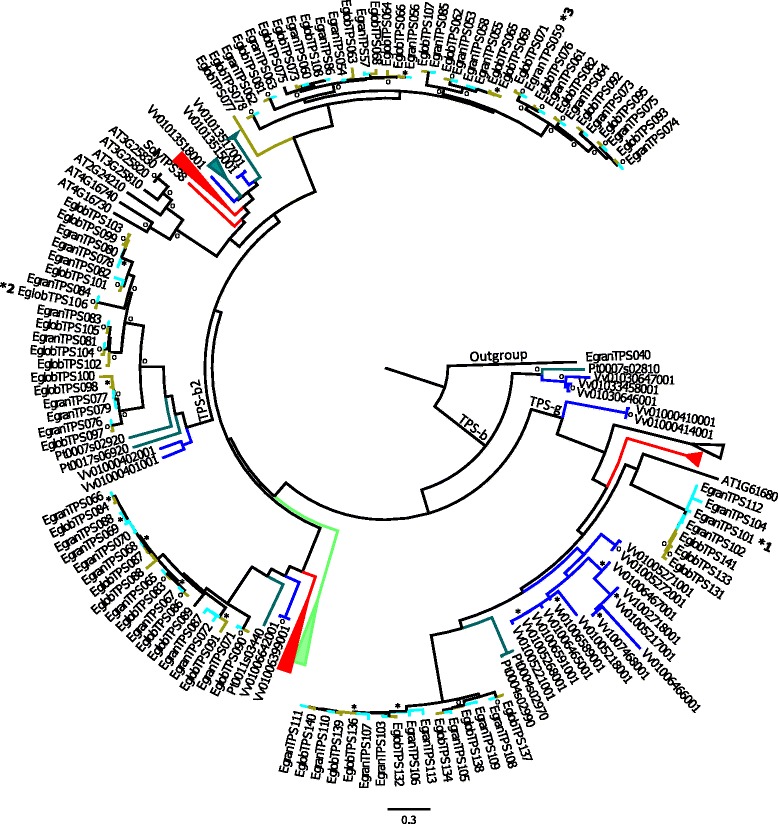
Phylogeny of the TPS-b and -g subfamilies. Maximum likelihood analysis of TPS-b and -g subfamilies from *E. grandis* and *E. globulus* in comparison to *Vitis vinifera* and *Arabidopsis thaliana*. Functionally characterized enzymes are indicated with an * with main products: *1 β-pinene, *2 isoprene (characterized by [56]), *3 γ-terpinene. Bootstrap values supported by ≥ 80% are designated * while those with bootstrap values ≥ 95% are designated ^**O**^. A single TPS-a gene from *E. grandis* was selected as outgroup.

**Figure 7:**
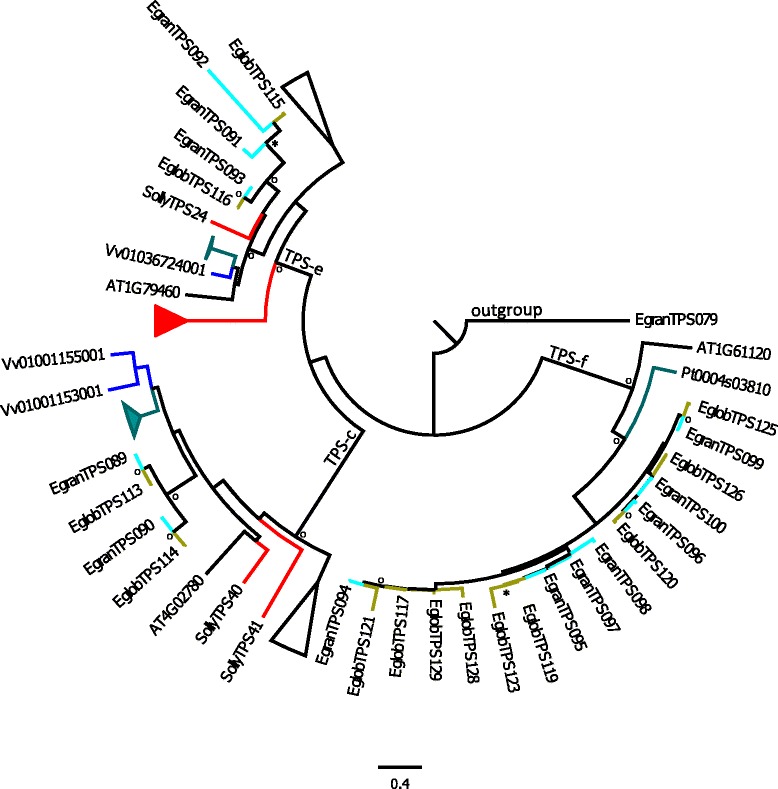
Phylogeny of the TPS-c, −e and -f subfamilies. Maximum likelihood analysis of TPS-c, −e and –f subfamilies from *E. grandis* and *E. globulus* in comparison to *Vitis vinifera* and *Arabidopsis thaliana*. Bootstrap values supported by ≥ 80% are designated * while those with bootstrap values ≥ 95% are designated ^**O**^. A single TPS-b gene from *E. grandis* was selected as outgroup.

The phylogenetic analysis shown here includes all known TPS genes from *Arabidopsis thaliana*, *Populus trichocarpa*, *Vitis vinifera* (cultivar Pinot Noir), *Solanum lycopersicum, Oryza sativa, Sorghum bicolor, Eucalyptus grandis* and *E. globulus.* The phylogenies are divided into groupings of (i) TPS-a (Figure 5 and Additional file 2: Figure S1), (ii) TPS-b and -g (Figure 6 and Additional file 2: Figure S2) and (iii) TPS-c, −e and -f (Figure 7 and Additional file 2: Figure S3). The phylogenies contained too many terminal nodes to be easily viewable (210 for (i), 161 for (ii)) and the full phylogenies are therefore presented in the supplemental files, while the figures show only TPS genes from *Arabidopsis thaliana*, *Vitis vinifera* and *Populus trichocarpa* in comparison to both eucalypt species studied here.

For the TPS-a subfamily, the most closely related genes to the eucalypt TPS clade are from *Vitis vinifera* and *Populus trichocarpa* (Figure 5). The *Eucalyptus* TPS genes form a paralogous cluster, which is different from some of the *Populus*/*Vitis* TPS-a clades (Figure 2, Additional file 2: Figure S1). A comparison of the two eucalypt species shows that 31 out of 52 genes are found in orthologous pairs for *E. grandis* (Additional file 1: Table S1) and 31 out of 45 for *E. globulus*. For several genes in *E. grandis*, the *E. globulus* ortholog is missing and *vice versa* (Figure 5). There are also several gene duplication events that post-date the separation of the two species. It is notable that the *Arabidopsis* TPS genes have longer branches than both grape and eucalypts suggesting a longer period of gene differentiation without novel gene duplication events.

Genes from TPS subfamilies –b and – g are shown in Figure 6 and Additional file 2: Figure S2. TPS-g forms a clade embedded within TPS-b. Eucalypts have two clades of TPS-g genes, one closely related to the only *Arabidopsis* TPS-g gene (At1g61680) gene and the other closest to two poplar genes. Six out of 13 and 10 TPS-g genes are in orthologous pairs for *E. grandis* and *E. globulus*, respectively (Additional file 1: Table S1), with several gene duplication events in the clade close to At1g61680 since the separation of these two eucalypt species.

The TPS-b subfamily falls into three separate clades, all of which contain multiple species (Additional file 2: Figure S2). The middle clade contains putative isoprene/ocimene synthases, and is described as TPS-b2, while the other two clades are described as TPS-b1 and contain putative cyclic monoterpene synthases (Figure 6). For the TPS-b1 19 out of 27 and 28 for *E. grandis* and *E. globulus*, respectively, occur in orthologous pairs (Additional file 1: Table S1). Roughly half of the isoprene/ocimene synthases occur in orthologous pairs (5/9 and 5/10 for *E. grandis* and *E. globulus*, respectively) with several gene duplications and loss of function events post- speciation (Additional file 1: Table S1).

Subfamilies TPS–c, −e and –f are involved in the formation of di-terpenes as well as mono- and sesquiterpenes. In the TPS-c subfamily, two out of two genes occur in orthologous pairs, while in TPS-e, two orthologous pairs are found, but *E. grandis* contains one extra gene (Figure 7, Additional file 1: Table S1). Gene copy numbers are very similar in these two families compared to other species (Table 1, Figure 7). However, there has been a significant expansion of the TPS-f family in eucalypts compared to other plant species. Only three orthologous pairs occur between the two eucalypt species out of seven and nine genes (Additional file 1: Table S1). Recent duplications (e.g. EglobTPS119 and EglobTPS123) as well as loss of function (e.g. the *E. globulus* orthologue of EgranTPS098) of genes have occurred.

### Functional characterization of full-length *Eucalyptus grandis* TPS genes

**Figure 8:**
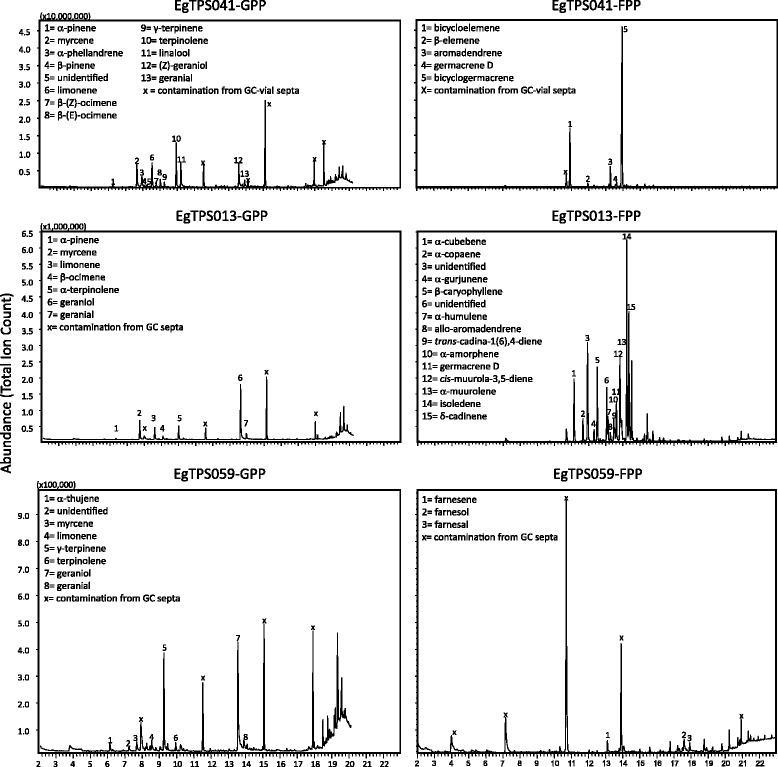
Gas chromatographic profile of the products formed *in vitro* by the enzyme activities of several *Eucalyptus grandis* TPS genes. Enzymes were incubated with GPP and FPP and products were analyzed as described in the Methods.

Of the nine genes synthesised for characterization studies, five have predicted transit-peptide sequences at the N-terminus, which suggests they are monoterpene synthases. Based on sequence characteristics, the remaining four genes are likely to be sesquiterpene synthases. We were able to express all nine genes in the vector, however, only three were able to produce significant amounts of terpenes and two produced traces of terpenes (Additional file 1: Table S5). We characterised a bicyclogermacrene synthase (EgranTPS041), an isoledene synthase (EgranTPS013) and a γ-terpinene synthase (EgranTPS059), all of which are multi-product enzymes which produced between 5 and 15 terpenes (Figure 8).

## Discussion

*Eucalyptus* is widely known for its foliar terpenes and the diversity of eucalypts in Australia is matched by a large diversity of terpenes in different species. Our analysis of two *Eucalyptus* genomes shows that this diversity arises from the largest gene family of terpene synthases yet described. More than 2000 papers have been published on *Eucalyptus* terpenes over the past century and they have been implicated in many processes within the plant, within the ecosystem and as large-scale influences on the environment via their impact on forest fires and in the atmosphere. The discovery of the diversity of TPS genes in the genome of *Eucalyptus* as well as recent discoveries of the control of quantitative variation and new evolutionary analyses of Myrtaceae terpenes [15] provides the first opportunity to understand how variation in *Eucalyptus* terpenes arises and how they can be manipulated in the world’s most widely planted hardwood tree.

Both *Eucalyptus grandis* and *E. globulus* have approximately four times as many putative functional TPS genes as *Arabidopsis thaliana*, three times as many as *Populus trichocarpa* and twice as many TPS genes as *Vitis vinifera*. Conifers may also have a large and diverse TPS gene family given the diversity of TPS genes that have been characterized [25], but despite recent assemblies of conifer genome sequences [26,27], accurate estimates of gene copy numbers are not available. Amongst the species that have been sequenced, the larger gene families are associated with species that have specialized storage organs for terpenes including *Eucalyptus* and grape [18]. No studies have reported on the sub-cellular localization of terpene synthases in eucalypts but they are presumed to occur in cells of the secretory cavities where the terpenes are stored along with other non-volatile constituents such as oleuropeyl glucose esters [28,29].

When comparing the branch lengths on the TPS phylogenies between each of the species in this study, it is clear that species with overall low numbers of TPS genes have longer branch lengths and those with large TPS gene families have shorter branch lengths. Species with a small TPS gene family such as *Arabidopsis thaliana* have few genes with long branches. This is especially apparent in the TPS-a subfamily where *Arabidopsis* has a large number of genes (Figure 5 and Additional file 2: Figure S1). *Eucalyptus* and *Vitis* have comparably small branch lengths, indicating rapid ongoing evolution as compared to *Arabidopsis*.

All but one study of the terpenes of *E. grandis* have focused on the foliar oils of the mature leaf. The profiles are complex with one study [30] detecting 99 components by capillary gas chromatography and the remainder between 38 and 67 [31-35]. The monoterpene fraction is dominated by either α-pinene or 1,8-cineole, which may be indicative of the occurrence of two terpene chemotypes in *E. grandis*. Since these two monoterpenes arise from different carbocations, it is likely that they are products of different TPS. However, none of the TPS genes that we characterized produced large amounts of either compound.

All published studies show a complex mixture of up to 30 sesquiterpenes with bicyclogermacrene and spathulenol usually dominant, but with a large diversity of other sesquiterpenes that were minor components of the oil. We characterized a bicyclogermacrene synthase that also produced a further four compounds and a second sesquiterpene synthase that produced 15 sesquiterpenes. Thus it may not require a large number of sesquiterpene synthases to be expressed in mature leaf to explain the complex oil profiles described previously.

Given the abundance of secretory cavities and high levels of terpene (monoterpene and isoprene) emissions from leaves of *Eucalyptus* it was not surprising that the largest proportion of *E. grandis* TPS genes are highly expressed in ‘green tissues’ (mature and young leaf but also floral buds and shoot tips). All of the TPS subfamilies found highly expressed in ‘green tissues’ belong to the TPS-a, −b1 and -b2 family, producing mono-, sesqui- and hemiterpenes as well as three acyclic terpene synthases from subfamily -g. However, we also found a significant group of TPS that were expressed in roots, xylem and phloem. Early studies identified secretory cavities in bark (including the root bark) the stem pith, and phloem, depending on the species [36] but the few studies of the essential oils in these tissues suggest that they are largely non-terpenoid [37]. Two of three samples cluster closely together in all tissues, which was not surprising as these trees derive from clonal material. The third sample, however, was a half-sib of the other two and showed some differences in gene expression.

The root-specific cluster of TPS genes that we identified may be involved in allelopathic interactions [7] and/or, as in other plants, in mediating interactions with herbivores and other soil organisms [38]. A further possibility is that root-derived terpenes may play a role in mycorrhizal colonization which is essential for many species of *Eucalyptus* [39,40]. Clearly there is a need for a broad investigation of terpenes in roots of eucalypts from both the chemical and molecular perspectives.

One possible explanation for the large number of TPS genes in *Eucalyptus* is that many are only involved in inducible responses. For example, Danner and co-workers isolated three TPS from *Populus* that were only expressed after gypsy moths had fed on the plants [41]. Other studies have identified TPS genes that are only expressed on treatment by methyl jasmonate [42]. However, the evidence for induced production of terpenes in *Eucalyptus* is mixed. The only comprehensive study involving methyl jasmonate found no evidence of an increased concentration of foliar terpenes or terpene phloroglucinol adducts in clones of a hybrid *E. grandis* × *E. camaldulensis*. Henerey and co-authors [24] argued that the induced foliar responses seen in deciduous trees and pines should not be expected in evergreen broadleaved trees like eucalypts because herbivory is much harder to predict in time and space. Furthermore, the terpene storage organs in eucalypts are primarily formed during leaf development [22], so induction may be difficult because the size and numbers of oil glands may not be very plastic.

There is however some evidence of induction of terpenes in woody tissues. Eyles *et al.,* [6] found significant concentrations of terpenes in new phloem of wound tissue of *E. globulus* and observed traumatic secretory cavities in the wood of *E. globulus* after pruning and fungal attack [43]. They argued that these inducible terpenes played a broad role in defence against infection after any damage to the phloem, either mechanical or biological. Our observations of a diverse range of TPS expressed in the phloem and wood tissues supports Eyles *et al.*’s [43] call for further investigation of terpenes in these tissues.

Further studies in eucalypts should focus on expression of terpene pathway genes including TPS after insect feeding, fungal infection and jasmonate treatments in a range of tissues and such studies may reveal a more complex pattern of induction. Such studies would help identify which (if any) of the TPS genes in the genome are inducible. This may help to explain the large size of the gene family.

A group of terpenoid compounds have been present since the evolution of the genus *Eucalyptus* ca. 50 million years ago and Keszei *et al.* [44] argued based on a phylogeny of TPS fragments from more than 20 species that early divergence and maintenance of function has suggested a closer correlation between sequence homology and function than seen in other plant groups. The comparison of TPS genes between *E. grandis* and *E. globulus* enables us to estimate both recent evolutionary activity of the terpenes synthase family (during the last 12 million years) and distant evolutionary activity. The results show that most TPS genes in *E. grandis* have evolved more than 12 million years ago, but also that there is still ongoing evolution as indicated by novel gene duplication and loss of function events. There are broad differences in the amount of conservation *vs*. evolution between TPS subfamilies and even within clades of the larger subfamilies. TPS-g and -f have the lowest proportion of orthologous pairs with 46 and 43%, respectively, while TPS-b and -c have the highest proportion with 82 and 100%, respectively. This shows that genes in subfamilies -b and -c are highly conserved. Overall, the similarities between gene families in the two species are very high and it would be interesting to compare further species pairs or even groups of *Eucalyptus* species to learn more about gene evolution in this gene family. Recent advances in DNA sequencing have enabled a large number of whole-genome sequencing projects and if a closely related species has a well-assembled and annotated genome, gene families can easily be characterized from such species. Species from genera like *Arabidopsis* and *Populus* are good candidates.

Isoprene is the smallest terpenoid compound, but has a significant impact on the planet’s atmosphere [45] through enhancement of aerosol and ozone formation. The physiological functions of isoprene in plants include heat protection [11,46], ozone tolerance [47] and tolerance of reactive oxygen species [48]. The biosynthetic cost for each isoprene molecule is very high (including at least 20 ATP and 14 NADPH), so the benefits for plants that emit large amounts such as *Eucalyptus* must be significant. Isoprene is the major component of eucalypt hydrocarbon emissions (64-100%) with the remainder being monoterpenes. *Eucalyptus grandis* and *E. globulus* are among the highest emitters of isoprene and hydrocarbons of any plant investigated to date [12] (although this has been challenged by Winters *et al.*, [49]). The ability to emit isoprene has evolved many times in plants and is argued to be a response to local environmental conditions [45]. Therefore, the high number of putative isoprene synthase genes found in *E. grandis* and *E. globulus* could be reflective of the environment eucalypts evolved in, though functional characterizations of each gene are needed to ensure they do not encode functional ocimene synthases. Australia is the hottest and driest continent and isoprene emissions have been implicated in mitigating heat stress [46]. Clade TPS-b2 contains putative isoprene/ocimene synthases, there are two recent gene duplications in *E. globulus* (EglobTPS099 and EglobTPS103; EglobTPS098 and EglobTPS100) and three putative losses of function (orthologous gene is missing in other species in EgranTPS079, EglobTPS102 and EgranTPS078) indicative of rapid ongoing evolution. *Eucalyptus grandis* has a sub tropical to tropical distribution with high rainfall, while *E. globulus* is found in temperate regions of southern Victoria and lowland Tasmania, but at sites that can experience high temperatures and dry summers. Characterizing the *Eucalyptus* isoprene synthases should clarify their role in mediating local heat stress and climate adaptation.

Genomic clusters of genes that encode multiple steps of biosynthetic pathways for plant secondary metabolites are an emerging theme in plant biology [50,51]. These clusters which typically span 100 s of kb, can contain parts of, or entire biosynthetic pathways and their evolutionary origin and biological function is of wide interest. In this study we have focused on smaller genomic areas to investigate clusters of duplicated TPS genes and their evolutionary fate. Nevertheless, we found multiple genes that are or may be involved in terpene biosynthesis, such as isopentenyl pyrophosphate isomerase on a cluster with three putative functional TPS-b loci, five TPS-b pseudogenes and five methyltransferases that may be involved in further modifications of terpene structures. Future work could investigate the larger scale genomic surroundings of the TPS gene clusters. Clusters of homologous genes evolve through gene duplication events and are widespread for enzymes that diversify the plant’s defense. There are examples for both TPS genes [18] and other gene families such as Kunitz protease inhibitors involved in insect herbivory defense [52], nucleotide binding site-leucine-rich repeat genes involved in fungal disease resistance [53] or polyphenol oxidases involved in bacterial and fungal disease resistance [49]. Due to the evolutionary origin of tandem gene duplications, genes within clusters have higher similarity to each other than to genes that are not in the cluster. We observed that the amino acid similarity of genes within clusters were twice as high as those of the whole gene sub-family (Additional file 1: Table S6), confirming their evolutionary origin from unequal crossing-over as suggested by Ober [54].

It has been difficult to functionally characterize terpene synthases from the Myrtaceae for several reasons including high levels of plant secondary metabolites present in leaves. Few studies have been successful [44,55,56]. Here, we used an alternative approach to capture functional terpene synthases via de-novo gene synthesis rather than gene amplification from cDNA. Our success rate for genes without target peptide (all sesquiterpene synthases) was high (75%), with the one unsuccessful gene (EgTPS032) also showing comparably low levels of gene expression across all tissues (Additional file 1: Table S5). Our success rate with the characterization of genes that putatively contain transit peptides (acyclic and cyclic monoterpenes from subfamilies TPS-b and –g) was much lower at 40% and this is possibly due to errors in assigning the location/end of the transit peptide as well as exon-intron boundaries. All genes that we attempted to functionally characterize were expressed, mostly in green tissue, but also in xylem and roots (Additional file 1: Table S5).

Most of the investigations of terpenes in *Eucalyptus* have been driven by the prospects of extracting industrially useful products and the mature leaf is the most abundant source of terpenes in the plants. Nonetheless the focus on terpenes in mature leaf tissue of *Eucalyptus* has meant that the functional and ecological interpretations of their role have been skewed towards questions such as defence against insect and vertebrate herbivory [57-59]. In contrast, reports of terpenes in other tissues such as roots and wound tissue [6] are sparse. Our results call for a re-interpretation of the role of terpenes in *Eucalyptus* since almost half the gene family is expressed in woody tissues and of those found in green tissues many are restricted to juvenile leaf. This suggests that there are many important functions of terpenes in eucalypts that have been underappreciated but which may be critical in explaining the success of eucalypts continent wide in Australia and in many other regions of the world where they have subsequently been cultivated.

## Conclusions

In this study, we have analysed the terpene synthase gene families from two *Eucalyptus* species, *E. grandis* and *E. globulus*. We investigated the genomic structure, gene diversity, evolutionary pathways and gene expression across seven tissues. High levels of intraspecific terpene diversity is reflected by the largest number of TPS genes found in a single species to date. Most of the TPS genes, as well as large numbers of TPS pseudogenes, are found in large genomic clusters which may originate from many gene duplication events leading to the terpene diversity found in the genus. Nearly half of the TPS genes were expressed in non-green tissues (root, phloem and xylem) and the role of terpenes in these tissues remains unknown. A phylogenetic analysis of the gene family revealed that most TPS clades have evolved little since the separation of the two *Eucalyptus* species, while a few clades show signs of recent evolution as shown by gene duplications and loss of function in one or both eucalypt species.

## Methods

### Discovery and annotation of putative terpene synthase genes in *Eucalyptus grandis*

The *Eucalyptus grandis* 8x BRASUZ1 genome (V1.1) sequence assembly (http://www.phytozome.net/eucalyptus.php) was used in a BLAST search based on conserved regions (Additional file 3) of all terpene synthase subfamilies with standard BLAST parameters. A preliminary list of genomic regions containing TPS genes was created and redundancies removed. In order to find full-length genes the up- and down-stream areas of the conserved regions were screened and a reverse BLAST search was used to confirm the identity of the putative TPS gene. Of the 172 loci that showed significant similarities to known TPS genes, 59 were excluded due to more than two frame shifts or stop codons in the translated gene. These pseudogenes are however of evolutionary interest and were therefore retained separately for later analysis of gene clusters (Additional file 1: Table S2). Loci with fewer than three exons (TPS genes generally contain between 7 and 13 exons depending on subfamily) were not included in the list, but those loci that did not cover the full gene length due to lack of assembled DNA sequence (falling off the scaffold) were included. Genes were manually aligned to known TPS genes from multiple species and sub-families (*Arabidopsis*: http://arabidopsis.org/, grape, tomato and *Backhousia citriodora* from Genebank (http://www.ncbi.nlm.nih.gov)) to determine exon-intron borders. After preliminary phylogenetic analysis of amino acid sequences (using Phyml [60]) putative functional genes were sorted into TPS subfamilies TPS-a, −b, −c, −e -f and -g by sequence similarity, then sorted by linkage group (LG) and then by position within LG. TPS-b was further divided into TPS-b1 for cyclic monoterpene synthases and TPS-b2 for putative isoprene/ocimene synthases. One characterised isoprene synthase from *Eucalyptus globulus* (AB266390) falls within this subgroup and is the synonym of EglobTPS106 [55]. Gene annotations were given from the first TPS-a gene (EgranTPS001) to the last TPS-g gene (EgranTPS113) in the assembled genome scaffolds (V1.1).

### Discovery of putative terpene synthase genes in *Eucalyptus globulus*

Illumina paired end shotgun short reads (75 bp) generated for a clonal genotype (X46) of *Eucalyptus globulus* genome (Department of Energy-Joint Genome Institute) were aligned against the *Eucalyptus grandis* genome as a reference using CLC Genomics Workbench (CLC, Aarhus, Denmark). Of the 224 million single short reads 215 million were matched (95.8%) against the reference sequence using default parameters for reference assembly in CLC Genomics Workbench. The remaining reads were assembled *de-novo* using standard parameters in CLC Genomics workbench into 5,739 contigs with an average length of 771 bp. Consensus sequences from both reference and de-novo assembly were imported into Geneious Pro (version 5.6.4, [61]) and BLAST searches with representatives from each subfamily of *E. grandis* TPS genes were performed. All hits with E values < e^−10^ were considered and redundancies removed. The genomic regions surrounding the BLAST hits (±2,500 bp) were investigated and reverse BLASTed against the non-redundant database at Genebank (http://www.ncbi.nlm.nih.gov). Coding regions for each gene were extracted and genes were examined for full length, stop codons and frame shifts. In total, 143 loci with high similarity to terpene synthase genes were discovered and 106 putative functional (full length genes with up to 2 frame shifts or stop codons) genes were considered for downstream analysis (Additional file 1: Table S3). Gene annotations were given in a similar way as in *E. grandis*, but were numbered through to include pseudogenes, thereby having a range from EglobTPS001 to EglobTPS143.

### Phylogenetic analysis of terpene synthase genes from *Eucalyptus* and other plants

Terpene synthase genes from *Arabidopsis thaliana* (http://arabidopsis.org/), *Populus trichocarpa* (http://www.plantgdb.org/PtGDB), *Vitis vinifera* (http://www.plantgdb.org/VvGDB/), *Solanum lycopersicum* [19], *Sorghum bicolor* (http://www.plantgdb.org/SbGDB/) and *Oryza sativa* (http://www.plantgdb.org/OsGDB/) were retrieved and divided by TPS subfamily. Amino acid alignments were made for subfamilies/groups of subfamilies, using ClustalW [62] in Genious Pro (version 5.6.4, [61]) using standard parameters. The alignments were then manually adjusted with a focus on diagnostic conserved regions such as the RLLR, DDXXD and NSE/DTE motifs. The alignment was then truncated to ensure that sites were homologous. All of exon 1 and parts of exon 2 were excluded due to high levels of variation, resulting from the presence of chloroplast target peptides in some genes and generally high levels of diversity. To create a phylogeny, we first tested which amino acid substitution model provided the maximum likelihood tree with the best AICc value (Akaike’s information criterion value, corrected for samples size) and further tested whether gamma distribution estimation and/or proportion of invariable sites estimation improved the AICc value using Phyml [60]. The amino acid substitution models that we tested were: EHO, EX2, EX3, JTT, LG, UL2, UL3 and WAG. The tree with the highest AICc value was using the JTT model with estimation of invariable sites and estimation of gamma distribution. The phylogeny of the EgranTPS gene family was determined using 100 bootstrap replicas. The phylogenies were visualized using FigTree v1.3.1 [63].

### RNA isolation and identification of terpene synthase transcripts in seven tissue types of *Eucalyptus grandis*

RNA was isolated from shoot tips, young leaves, mature leaves, floral buds, immature xylem and phloem tissues collected from three actively growing *E. grandis* trees as described previously [64]. Root tissues were sampled from rooted cuttings of the same *E. grandis* genotypes. Sampled tissues were immediately frozen in liquid nitrogen and stored at −80°C until RNA extraction. Total RNA was isolated as described previously [65] and used for Illumina RNAseq analysis at the Center for Genome Research & Biocomputing at Oregon State University. The RNA read pairs (50 bp paired end) were mapped to predicted gene models in the draft *E. grandis* genome sequence (Department of Energy-Joint Genome Institute V1.0, http://www.phytozome.net) using TopHat version 1.3 [66] and regions which were identified as terpene synthase genes. Gene expression values (fragments per kilobase of coding region per million mapped fragments, FPKM) were calculated for each predicted locus using Cufflinks version 1.1.0 [67].

### Analysis of transcript abundance and cluster analysis of genes and tissues

For each of the 21 RNAseq datasets (seven different tissues in triplicate, deposited at the *Eucalyptus* Genome Integrative Exploreer: http://eucgenie.org/), the log2 of the FPKM value was used to create a heat map in Expander (version 6.0) (EXPression ANalysis and DisplayER; Ron Shamir’s Computational genomics group, University of Tel Aviv, Israel). Hierarchical cluster analysis for both tissues and genes was performed in GenStat (version 14.1) with Euclidian matrix formation and the complete linkage method.

### Functional characterization of recombinant proteins

From the list of putative functional terpene synthases from *E. grandis*, we took only those that were full length and had no frame shift mutations or premature stop codons. We focussed on the terpene synthases highly expressed in leaves, since these are likely to be the most important in defence against folivores. We chose between one and two sequences from each group within the TPS-a, −b and -g subfamilies; a total of 9 sequences (Additional file 1: Table S5). The coding sequences minus putative chloroplast target peptide region were synthesised (quality: research grade) and inserted into the pUC57 vector (Genescript, NJ).

We cloned the coding sequences into the pASK-IBA37+ expression vector (IBA GmbH, Germany), using methods described by Köllner *et al.* [68,69]. Briefly, the coding sequences were amplified from the pUC57 vector using primers with overhangs complimentary to the vector overhangs. The amplicon was ligated into the pASK-IBA37+ vector; these constructs were introduced into TOP10 *E. coli* cells (Invitrogen, CA) and fully sequenced to confirm the absence of PCR-induced errors. Liquid cultures were grown at 37°C to OD_600_ between 0.5 and 0.6. The expression of pASK-IBA37+ constructs in TOP10 cells was induced with anhydrotetracycline (final concentration 200 μg.l). After 20 h incubation at 18°C, the cells were collected by centrifugation and disrupted by a 4 × 30 s treatment at 50% with a sonicator (Branson Sonifier 250, Germany) in chilled extraction buffer (50 mM Tris–HCl pH 7.5, 10% (v/v) glycerol, 5 mM MgCl_2_, 5 mM dithiothreitol, 5 mM Na-ascorbate pH 7.0, 0.5 mM phenylmethylsulfonyl fluoride). The cell fragments were removed by centrifugation at 14,000 × g, and the supernatant was desalted into the assay buffer (10 mM Tris–HCl pH 7.5, 10% glycerol (v/v), 1 mM dithiothreitol) by passage through a Econopac 10DG column (Bio-Rad, CA).

Standard assays containing 30 μL of the bacterial extract in assay buffer with 13.2 ng/μl GPP or (E,E)-FPP, 10 mM MgCl_2_ and 58 μl of assay buffer (10 mM Tris–HCl, 10% glycerol, 1 mM DTT; pH 7.5), in a glass gas chromatograph assay tube (Macherey-Nagel, Germany). A solid phase microextraction fibre consisting of 100 μm polydimethylsiloxane (SUPELCO, PA) was placed into the headspace of the tube during a 45 min incubation at 35°C. The solid phase microextraction fibre was then directly inserted into the injector of a gas chromatograph for product analysis.

A Shimadzu model GC-2010 gas chromatograph was employed with the carrier gas hydrogen at 1 ml·min^−1^, splitless injection (injector temperature: 220°C) with an EC-5 column (5% phenyl-methylpolysiloxane, 30 m × 0.25 mm i.d. × 0.25 μm film thickness, (Grace, Deerfield, IL)). The temperature program for monoterpenes was from 50°C (3-min hold) to 150°C at 7°C·min^−1^, then to 300°C (2 min hold) at 100°C·min^−1^; and for sesquiterpenes from 80°C (3 min hold) to 200°C at 7°C·min^−1^, then to 300°C (2 min hold) at 100°C. min^−1^. Terpenes were detected with a mass spectrometer (GCMS-QP 2010 Plus, Shimadzu), which was coupled to the gas chromatograph. Terpenes were identified with the Shimadzu software “GCMS Postrun Analysis” with the mass spectral library “Wiley8” (Hewlett Packard, Palo Alto).

### Availability of supporting data

The data sets supporting the results of this article are included within the article and its additional files.

## Additional files

Additional file 1:
**This file contains 6 supplemental tables.**
Additional file 2:
**This file contains 3 supplemental figures.**
Additional file 3:
**This file contains 2 supplemental text documents.**

## Abbreviations

TPS: Terpene synthase
MEP: Methylerithritol phosphate pathway
MVA: Mevalonate pathway
GPP: Geranyl pyrophosphate
GGPP: Geranylgeranyl pyrophosphate
FPP: Farnesyl pyrophosphate
LG: Linkage group
AICc: Akaike’s information criterion value, corrected for samples size
FPKM: Fragments per kb of coding region per million mapped fragments
EST: Expressed sequence tag
QTL: Quantitative trait loci
