# Supplementary material for: The Eucalyptus terpene synthase gene family
Source: BMC Genomics. 2015 Jun 11;16(1):450. doi: 10.1186/s12864-015-1598-x (PMC4464248; doi:10.1186/s12864-015-1598-x)
Supplement: Additional file 2: — This file contains 3 supplemental figures. [file 12864_2015_1598_MOESM2_ESM.pdf]

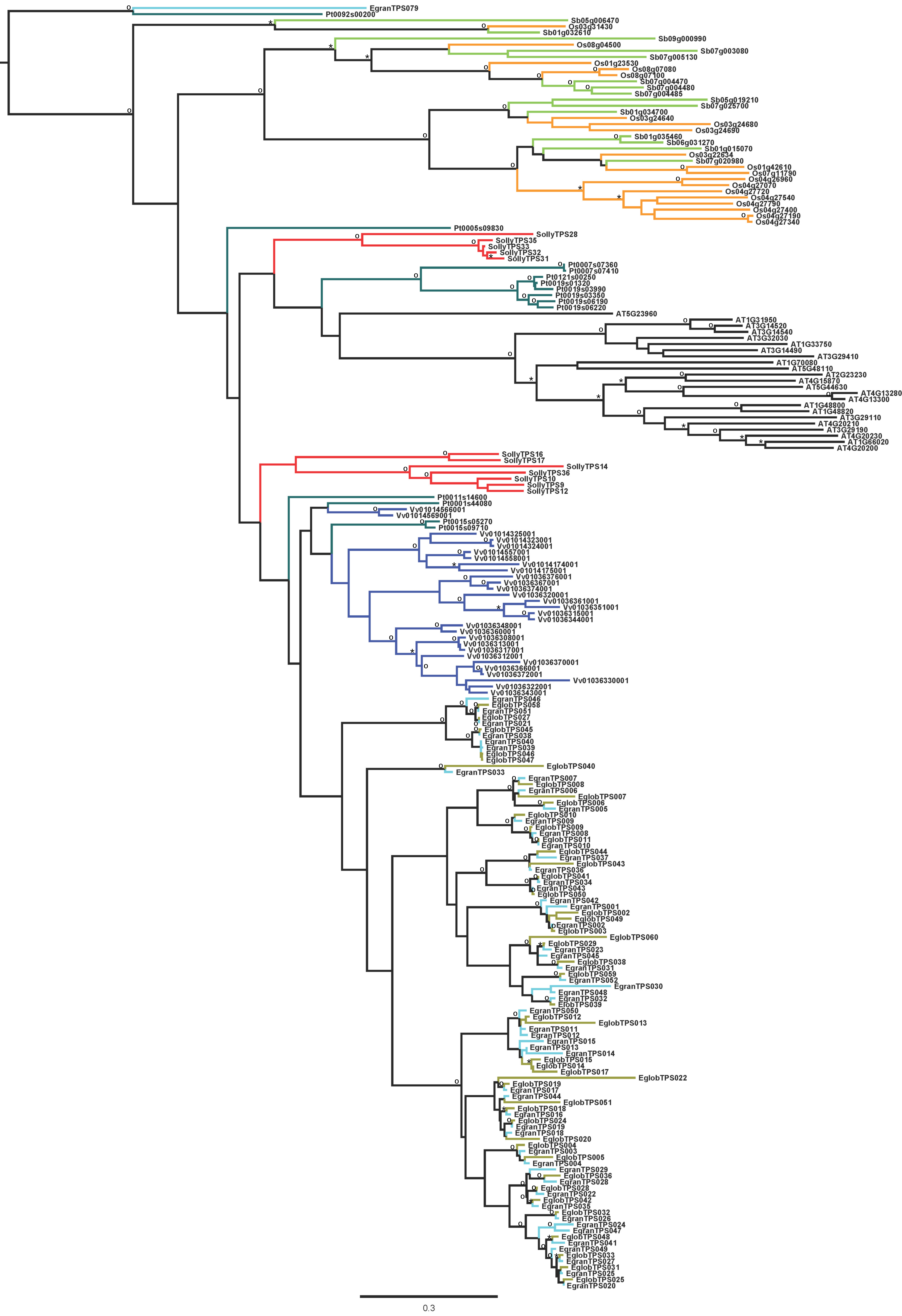

Figure S1: Maximum likelihood analysis of TPS-a subfamily from *E. grandis* and *E. globulus* in comparison to *Solanum lycopersicum*, *Oryza sativa*, *Populus trichocarpa*, *Sorghum bicolor*, *Vitis vinifera* and *Arabidopsis thaliana.* Bootstrap values supported by ≥ 80% are designated * while those with bootstrap values ≥ 95% are designated **o**.

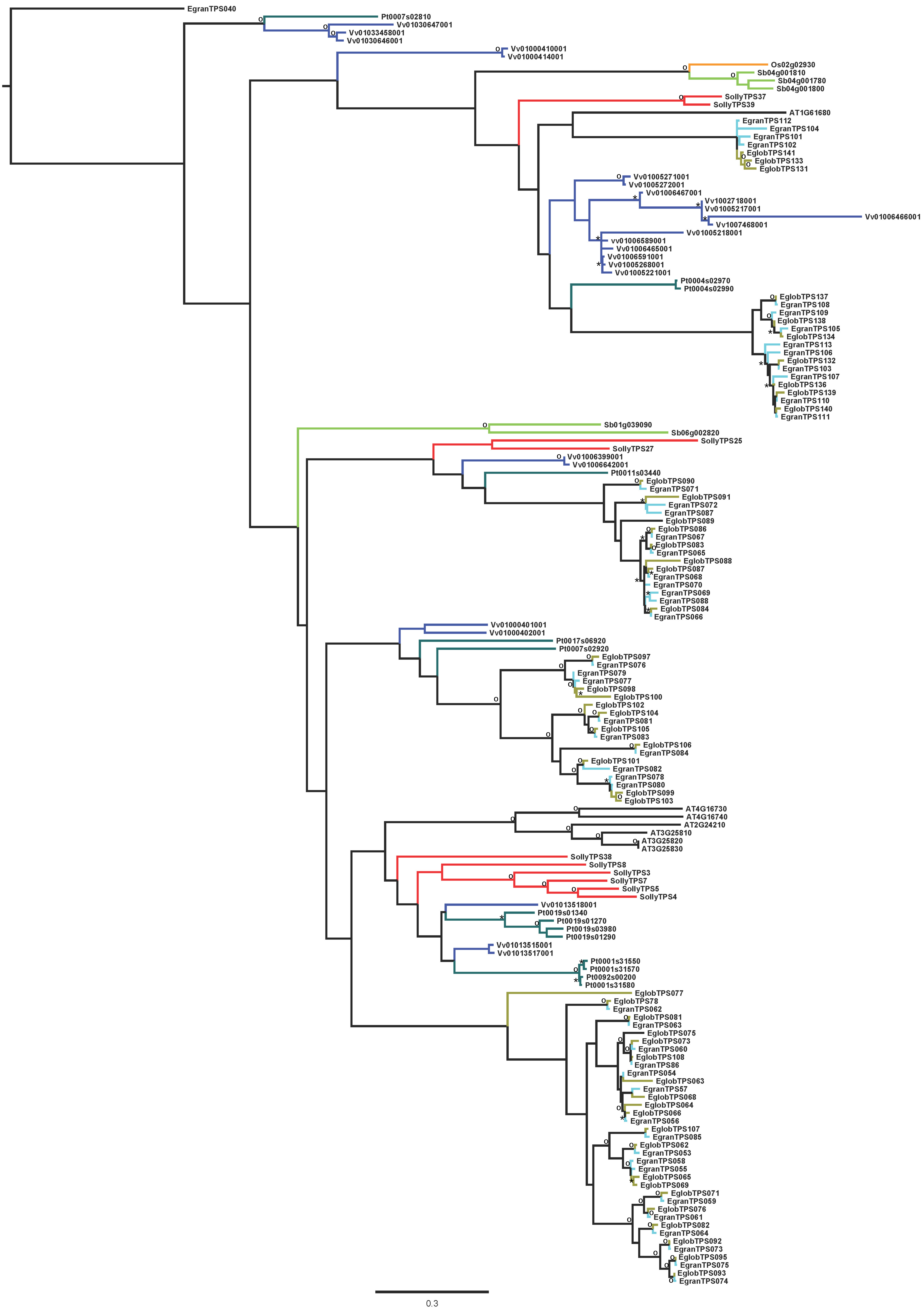

Figure S2: Maximum likelihood analysis of TPS-b and -g subfamilies from *E. grandis* and *E. globulus* in comparison to *Solanum lycopersicum*, *Oryza sativa*, *Populus trichocarpa*, *Sorghum bicolor*, *Vitis vinifera* and *Arabidopsis thaliana.* Bootstrap values supported by ≥ 80% are designated * while those with bootstrap values ≥ 95% are designated o.

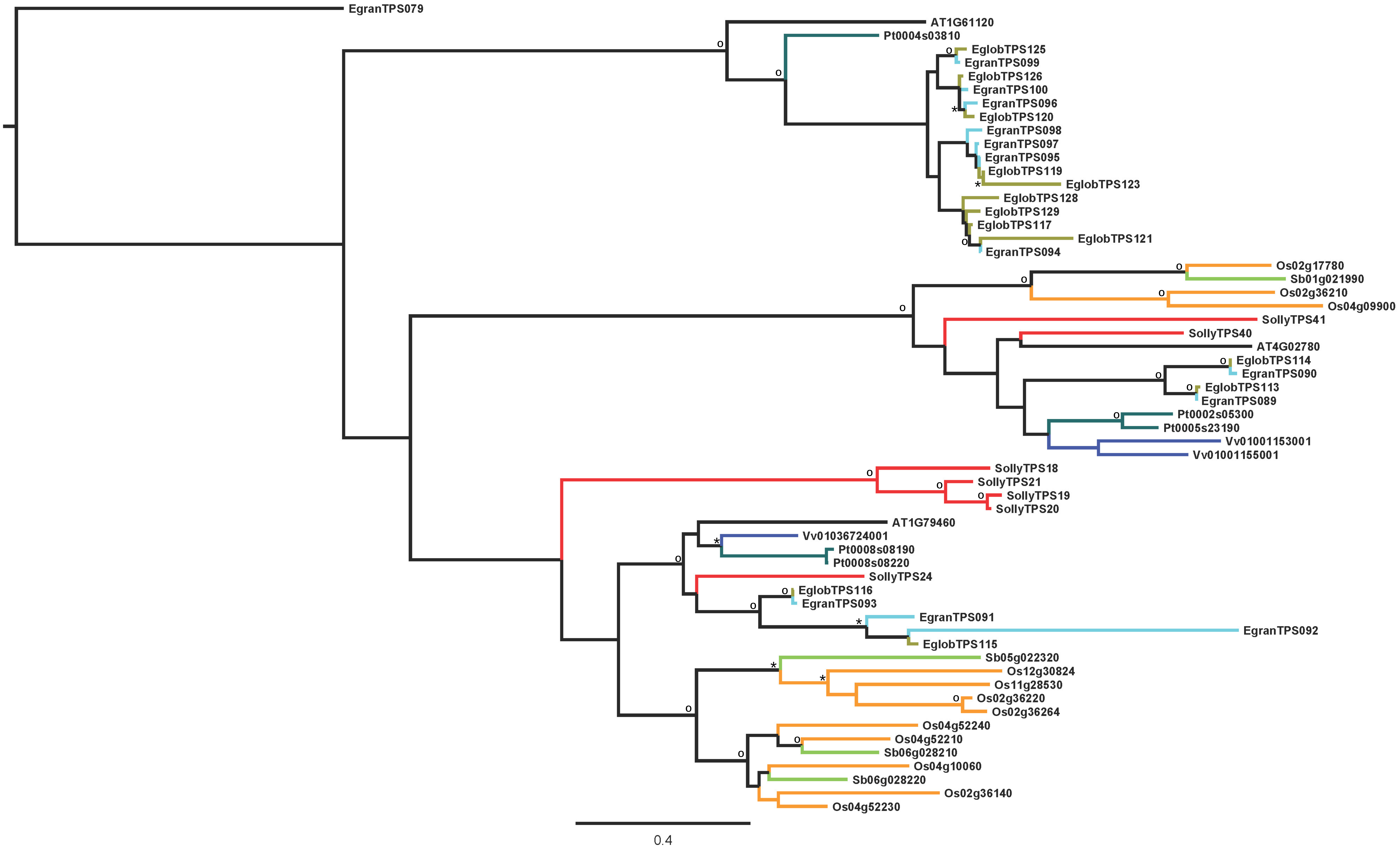

Figure S3: Maximum likelihood analysis of TPS-c, -e and -f subfamilies from *E. grandis* and *E. globulus* in comparison to *Solanum lycopersicum*, *Oryza sativa*, *Populus trichocarpa*, *Sorghum bicolor*, *Vitis vinifera* and *Arabidopsis thaliana.* Bootstrap values supported by ≥ 80% are designated * while those with bootstrap values ≥ 95% are designated o.
